# Proteomic analysis of holocarboxylase synthetase deficient-MDA-MB-231 breast cancer cells revealed the biochemical changes associated with cell death, impaired growth signaling, and metabolism

**DOI:** 10.3389/fmolb.2023.1250423

**Published:** 2024-01-11

**Authors:** Witchuda Sukjoi, Clifford Young, Mitchell Acland, Siraprapa Siritutsoontorn, Sittiruk Roytrakul, Manuela Klingler-Hoffmann, Peter Hoffmann, Sarawut Jitrapakdee

**Affiliations:** ^1^ Department of Biochemistry, Faculty of Science, Mahidol University, Bangkok, Thailand; ^2^ Clinical and Health Sciences, University of South Australia, Adelaide, SA, Australia; ^3^ Adelaide Proteomics Centre, School of Biological Sciences, The University of Adelaide, Adelaide, SA, Australia; ^4^ Functional Proteomics Technology Laboratory, National Center for Genetic Engineering and Biotechnology, National Science and Technology Agency, Pathumthani, Thailand

**Keywords:** holocarboxylase synthetase, biotin carboxylases, breast cancer, proteomics, metabolism, breast cancer

## Abstract

We have previously shown that the holocarboxylase synthetase (HLCS) is overexpressed in breast cancer tissue of patients, and silencing of its expression in triple-negative cancer cell line inhibits growth and migration. Here we investigated the global biochemical changes associated with HLCS knockdown in MDA-MB-231 cells to discern the pathways that involve HLCS. Proteomic analysis of two independent HLCS knockdown cell lines identified 347 differentially expressed proteins (DEPs) whose expression change > 2-fold (*p* < 0.05) relative to the control cell line. GO enrichment analysis showed that these DEPs were mainly associated with the cellular process such as cellular metabolic process, cellular response to stimulus, and cellular component organization or biogenesis, metabolic process, biological regulation, response to stimuli, localization, and signaling. Among the 347 identified DEPs, 64 proteins were commonly found in both HLCS knockdown clones, confirming their authenticity. Validation of some of these DEPs by Western blot analysis showed that plasminogen activator inhibitor type 2 (SerpinB2) and interstitial collagenase (MMP1) were approximately 90% decreased in HLCS knockdown cells, consistent with a 50%–60% decrease in invasion ability of knockdown cells. Notably, argininosuccinate synthase 1 (ASS1), one of the enzymes in the urea cycle, showed approximately a 10-fold increase in the knockdown cells, suggesting the crucial role of HLCS in supporting the urea cycle in the triple-negative cancer cell line. Collectively, our proteomic data provide biochemical insights into how suppression of HLCS expression perturbs global changes in cellular processes and metabolic pathways, impairing cell growth and invasion.

## Introduction

Cancer cells exhibit several profound changes in cellular and biochemical pathways that enable them to grow and invade other tissues or organs (Hanahan, 2022). Metabolic reprogramming constitutes one of the cancer hallmarks and has received much attention in recent years. Metabolic reprogramming is defined as the specific adjustment of metabolic pathways which supports biomass synthesis and bioenergetic requirements during oncogenic transformation. These changes include increased aerobic glycolysis (Warburg effect), pentose phosphate pathway activity, glutaminolysis, and *de novo* synthesis of lipids, nucleotides, and amino acids (Pavlova and Thompson, 2016; Vander Heiden and DeBerardinis, 2017), all of which are regulated by p53, c-Myc, and HIF-1α (Dang et al., 2008; DeBerardinis and Chandel, 2016). Therefore, inhibiting these metabolic pathways by small molecules or enzyme inhibitors hold promise for novel anti-cancer drugs (Vander Heiden, 2011; Stine et al., 2022).

The biotin-dependent carboxylases (BDCs), including acetyl-CoA carboxylase 1 and 2 (ACC1 and ACC2), methylcrotonyl-CoA carboxylase (MCC), propionyl-CoA carboxylase (PCC), and pyruvate carboxylase (PC) play pivotal roles in various intermediary metabolism including lipogenesis, gluconeogenesis, odd-chain fatty acid catabolism, and branched-chain amino acid catabolism (Tong, 2017). All BDCs have been reported to be overexpressed in several types of human cancer (Milgraum et al., 1997; Phannasil et al., 2015; Sellers et al., 2015; Svensson et al., 2016; Liu et al., 2019; Ngamkham et al., 2020; Du et al., 2021; Gondas et al., 2022); only the pro-oncogenic roles of ACC1 and PC have been studied extensively. Pharmacological inhibition or genetic ablation of these enzymes attenuates cancer growth, invasion and metastasis (Phannasil et al., 2015; Sellers et al., 2015; Davidson et al., 2016; Svensson et al., 2016; Shinde et al., 2018; Stoiber et al., 2018). Although attenuating the activity or expression of BDC is ideal for inhibiting the growth of cancers, inhibiting the BDC regulator may be more effective. The holocarboxylase synthetase (HLCS) regulates BDC activity through the covalent attachment of a biotin moiety to the specific lysine residue at the C-terminus of the biotin carboxyl carrier domain, in a process known as biotinylation (Leon-Del-Rio et al., 2017; Sternicki et al., 2017). Inhibition of biotinylation abrogates the carboxylase activity, thus lowering all BDC activities simultaneously. We have previously shown that HLCS is upregulated in breast cancer tissues, and its expression is correlated with lymph node invasion and poor prognosis (Sukjoi et al., 2020). Elevated expression of HLCS in breast cancer tissues is believed to support biotin carboxylase activities required for oncogenic growth and metastasis (Sukjoi et al., 2020). Pharmacological inhibition of biotin incorporation into biotin carboxylases can inhibit HLCS activity, thus providing an alternative means to block cancer growth (Yoon et al., 2021; Siritutsoontorn et al., 2022). We have recently shown that suppressing HLCS expression in both low and highly-invasive breast cancer cell lines, MCF-7 and MDA-MB-231, respectively, inhibits their growth and migration, accompanied by cell cycle arrest, indicating the pro-oncogenic role of HLCS (Siritutsoontorn et al., 2022). Although the growth defect observed in both MCF-7 and MDA-MB-231 cells primarily results from the lowered carboxylase activities, it is unclear how the impaired carboxylase activities globally perturb biological pathways in breast cancer cells. Here we performed a proteomic analysis of HLCS knockdown MDA-MB-231 cells and demonstrated that suppression of HLCS expression perturbs various biological processes associated with cell survival and affects several key metabolic enzymes.

## Materials and Methods

### Holocarboxylase synthetase knockdown cells and maintenance

The two previously described HLCS knockdown MDA-MB-231 cell lines, KD 868 and KD 1950, and scrambled control cell lines were used in this study (Siritutsoontorn et al., 2022). They were cultured in Minimal Essential Medium (MEM) supplemented with 10% (v/v) fetal bovine serum (FBS) and 1% (v/v) penicillin/streptomycin. Cells were cultured at 37°C with 5% CO_2_ and routinely sub-cultured every 3 days.

### Clonogenic assay

Five hundred cells of HLCS knockdown and scrambled control cell lines were seeded into a 100-mm culture dish containing MEM supplemented with 10% (v/v) fetal bovine serum and maintained at 37°C with 5% CO_2_. Following 10 days, the culture media were removed, and the colonies were washed with phosphate buffer saline (PBS). Cells were fixed with 100% (v/v) methanol for 20 min at room temperature and gently washed with water. The colonies were stained with 0.5% (w/v) crystal violet in 25% (v/v) methanol for 45 min at room temperature in the dark, rinsed with water, air-dried overnight, and counted.

### Invasion assay

Approximately 1 × 10^6^ cells/mL were suspended in MEM media supplemented with 0.1% (v/v) BSA and incubated with 1 μg/mL of fluorogenic calcein-AM (Invitrogen) for 30 min at room temperature in the dark. Cells were then centrifuged at 1,500 rpm for 5 min, washed three times with MEM supplemented with 0.1% (v/v) BSA, and resuspended at the density of 8 × 10^5^ cells/mL with MEM containing 0.1% (v/v) BSA. Afterwards, 4 × 10^4^ cells were suspended in 49 mL of MEM before placing onto the filter (12-µm pore size) of 96-well ChemoTx^®^ chemotaxis system (Neuro Probe), which was pre-coated with Geltrex (Life Technologies). Cells were allowed to migrate from the filter to the bottom compartment containing 29 µL of MEM supplemented with 10% (v/v) FBS at 37°C for 6 h. The non-migrated cells on the top of the filter were gently removed with a paper towel before the fluorescence intensity generated from the calcein-labeled cells in the bottom compartment was measured with excitation and emission wavelength at 485 and 520 nm, respectively, in a Triad series multimode detector (Dynex Technologies).

### SDS-PAGE and Western blot analysis

Whole-cell lysates of HLCS knockdown and scramble MDA-MB-231 cells were extracted in 100 μL of 1x radio-immunoprecipitation assay (RIPA) buffer containing 50 mM Tris-HCl pH 7.4, 1% (v/v) NP-40, 0.25% (w/v) sodium deoxycholate, 150 mM NaCl, 1 mM EDTA, and 1x protease inhibitor cocktail (Roche) on ice for 30 min before centrifugation at 12,000 × g for 15 min at 4°C. Protein concentrations were determined using Bradford regent (Bio-Rad). Protein (30 μg) was separated on a 7.5% discontinuous SDS-PAGE mini gel using 1x glycine buffer (25 mM Tris-HCl pH 8.3, 193 mM glycine, 0.1% (w/v) SDS) under reducing conditions. The protein was transferred from polyacrylamide gel to polyvinylidene difluoride membranes by Trans-Blot®Turbo™ Transfer system (Bio-Rad) with 1x transfer buffer (25 mM Tris-HCL pH 8.3, 192 mM glycine, 0.1% (w/v) SDS and 20% (v/v) methanol) at constant 25 V for 20 min. Then, the membranes were blocked in a blocking buffer containing 5% (w/v) skim milk and 1% (v/v) Tween 20 in 1x PBS pH 7.4 at 4°C overnight. The blots were then incubated with appropriate primary antibodies: 5 µg of rabbit anti-HLCS antibody (Bailey et al., 2010), 1:1,000 dilution of rabbit anti-MMP1 polyclonal antibody (E9S9N) (Cell Signaling Technologies), or 1:5,000 dilution of rabbit anti-SerpinB2 polyclonal antibody (ab47742, Abcam) at room temperature for 2 h. Excess antibodies were washed four times with 1x PBS buffer containing 1% Tween 20 before incubating with 1:20,000 dilution of mouse anti-rabbit IgG conjugated with horseradish peroxidase (Dako) at room temperature for 1 h. The immunoreactive bands representing each protein were detected using a chemiluminescence substrate (Merck, Millipore). Following MMP1 detection, the blot was incubated with stripping buffer (20 mM glycine, 0.1% SDS and 1% Tween 20, pH 2.2) and blocked in a blocking buffer at room temperature for 1 h. The membrane was incubated with 1:1,000 dilution of rabbit anti-ASS1 polyclonal antibody (D4O4B) (Cell Signaling Technologies) at room temperature for 2 h. Then, the membrane was incubated with a secondary antibody and developed as above.

### Proteomic analysis

#### Sample preparation

The proteomics analysis was performed with three sample groups, including scrambled control and two HLCS knockdown cell lines: KD 868 and KD 1950. Each sample group was performed in three biological replicates, each with two technical replicates. HLCS knockdown cell lines were grown to 90% confluence in MEM supplemented with 10% (v/v) FBS and 1% (v/v) penicillin/streptomycin. Cells were trypsinized and harvested by centrifugation at 1,500 × g for 5 min. The supernatant was discarded, and the cell pellet was washed three times with PBS. The cell pellet was snap-frozen at −80°C and suspended in 200 μL of RIPA buffer supplemented with 1x protease inhibitor cocktail (Sigma-Aldrich). Cells were disrupted by passing cell suspension through a 26.5-gauge needle 5 times. The lysates were centrifuged at 20,000 × g for 30 min at 4°C, and the supernatant was collected. Four volumes of cold acetone (Chem-Supply) were added to the supernatant before storing at −20°C overnight. The proteins were precipitated by centrifugation at 20,000× g for 20 min at −9°C. The pellet was washed with 200 μL of cold acetone and dried on ice for 30 min before resuspending in 40 μL of 8 M urea (Merck) in 50 mM ammonium bicarbonate (Fluka Analytical). Protein concentration was determined by tryptophan fluorescence.

#### Trypsin digestion

Protein samples (100 μg) were subjected to disulfide bond reduction with 10 mM dithiothreitol (Sigma-Aldrich) and incubated at room temperature for 1 h. The samples were then alkylated with 15 mM 2-chloroacetamide (Sigma-Aldrich) at room temperature for 30 min in the dark before 10-fold diluted with 50 mM ammonium bicarbonate. Trypsin (Mass spectrometry grade, Promega) was added to the protein at a ratio of 1:50, and the digestion was performed at 37°C in a ThermoMixer (Eppendorf) with 500 rpm agitation for 18 h. Approximately 10 μL of formic acid (LC-MS grade, Merck) was added to each sample before centrifugation at 20,000 × g for 10 min. The supernatant was transferred to a new tube, and the peptides quantitated by tryptophan fluorescence. The resulting samples were desalted using a 1 cc Sep-Pak C18 cartridge (Waters). The stationary phase was flushed with 1 mL of methanol, 3 mL of 80% (v/v) acetonitrile in 0.1% (v/v) formic acid, and then 4 mL of 0.1% (v/v) formic acid to equilibrate the columns before the samples were loaded. The samples were washed with 3 mL of 0.1% (v/v) formic acid, eluted with 1 mL of 50% (v/v) acetonitrile in 0.1% (v/v) formic acid, and dried under vacuum centrifugation (Christ AVC 2–25 CD plus) at 50°C.

#### Liquid chromatography-mass spectrometry

LC-MS analysis was performed on an Ultimate 3000 RSLC-nano system connected to an Orbitrap Exploris 480 mass spectrometer (Thermo Scientific, Bremen, Germany). Approximately 1 μg of each peptide sample was resuspended in 0.1% (v/v) formic acid and loaded onto a 25 cm fused silica column heated to 50°C. The internal diameter (75 μm) of the column was packed with 1.9 μm C18 particles. Peptides were separated over 70 min with a linear gradient of 3%–20% acetonitrile in 0.1% formic acid at a flow rate of 300 nL/min. Compensation voltages (−50 and −70 V) were applied from a FAIMS Pro interface (Thermo Scientific) to regulate the entry of ionized peptides into the mass spectrometer. MS scans (*m/z* 300–1500) were acquired at resolution 60,000 (*m/z* 200) in positive ion mode, with MS/MS scans of fragment ions measured at 15,000 resolution after applying 27.5% higher-energy collision dissociation. A dynamic exclusion period of 40 s was specified.

### Data processing and bioinformatic analysis

Raw data was processed with Proteome Discoverer v2.4 (Thermo Scientific). Searches were performed against the human FASTA database using the SEQUEST HT search engine, with the precursor and fragment mass tolerances set to 10 ppm and 0.02 Da, respectively. Two missed cleavage sites were allowed, with the minimum peptide length specified at 6 amino acids. Oxidation (+15.995), deamidation (+0.984), N-terminal acetylation (+42.011), N-terminal methionine loss (−131.040) and N-terminal methionine loss and acetylation (−89.030) were included as variable modifications, and cysteine carbamidomethylation was included as a fixed modification. To understand the differentially expressed proteins (DEPs) involvement in various biological processes, molecular functions, and cellular components, a list of all DEPs was subjected to gene annotation analysis using PANTHER database analysis tool version 16.0 (http://www.pantherdb.org/) (Thomas et al., 2003).

### Statistical analysis

All experiment results are expressed as means ± S.D. The statistical analysis was performed using GraphPad Prism software (version 8.4.0; GraphPad Software, Inc., La Jolla, CA). Significant differences between the sample groups were calculated by one-way factorial analysis of variance (ANOVA), followed by Dunnett’s test. A *p*-value of <0.05 indicated statistically significant.

## Results

### Quantitative analysis of the proteome of holocarboxylase synthetase knockdown MDA-MB-231 cells

**FIGURE 1:**
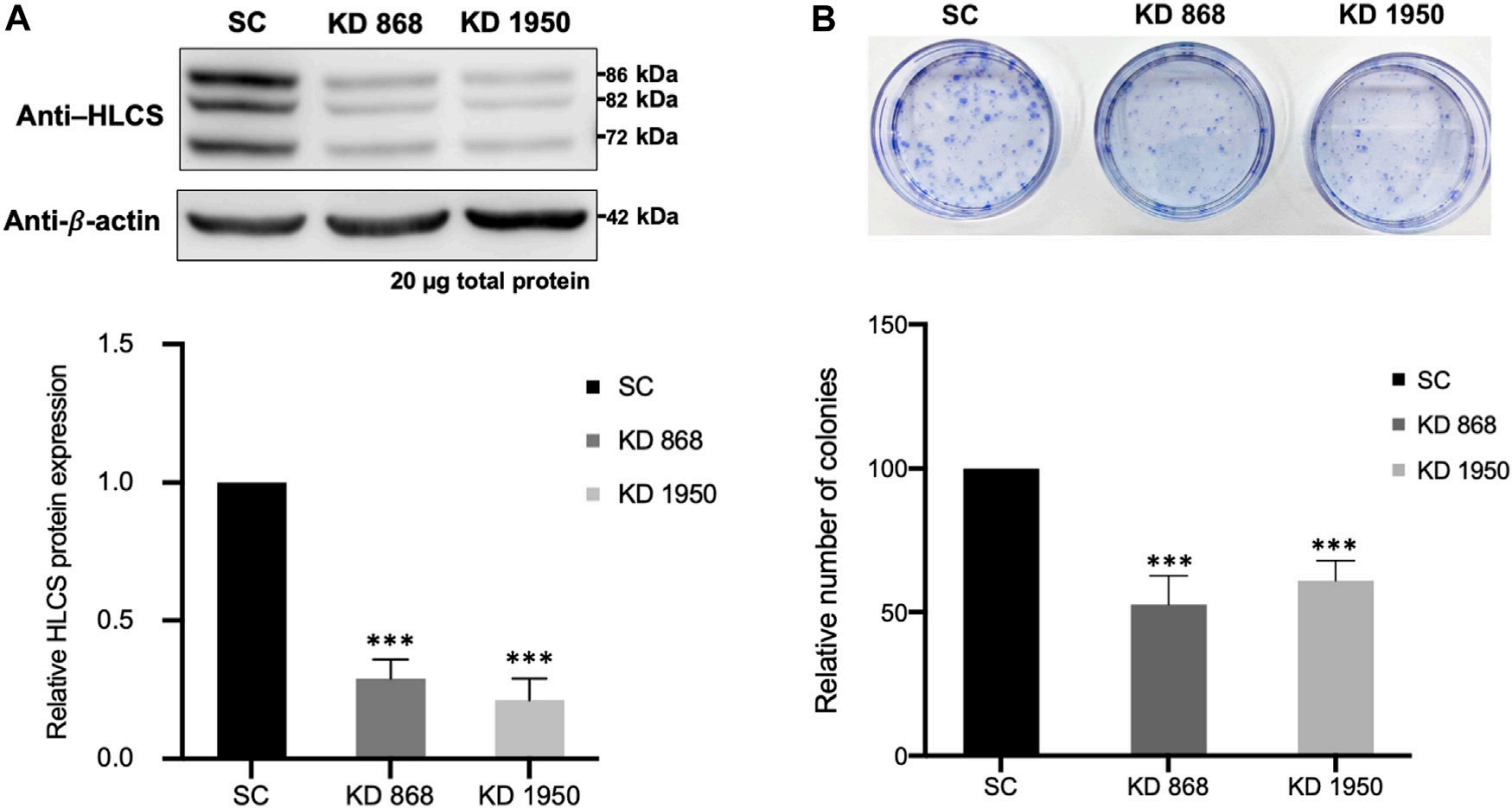
HLCS knockdown MDA-MB-231 cell lines show impaired clonogenic growth **(A)** Western blot analysis of HLCS protein in two HLCS knockdown clones, KD 868 and KD 1950 (top panel), and their expression level relative to that of the scrambled control cell line (SC) which was arbitrarily set to 1 (bottom panel). **(B)** Crystal violet staining of HLCS knockdown colonies following 10 days of clonogenic growth (top panel) and the relative number of clones (bottom panel). ***p < 0.001.

**FIGURE 2:**
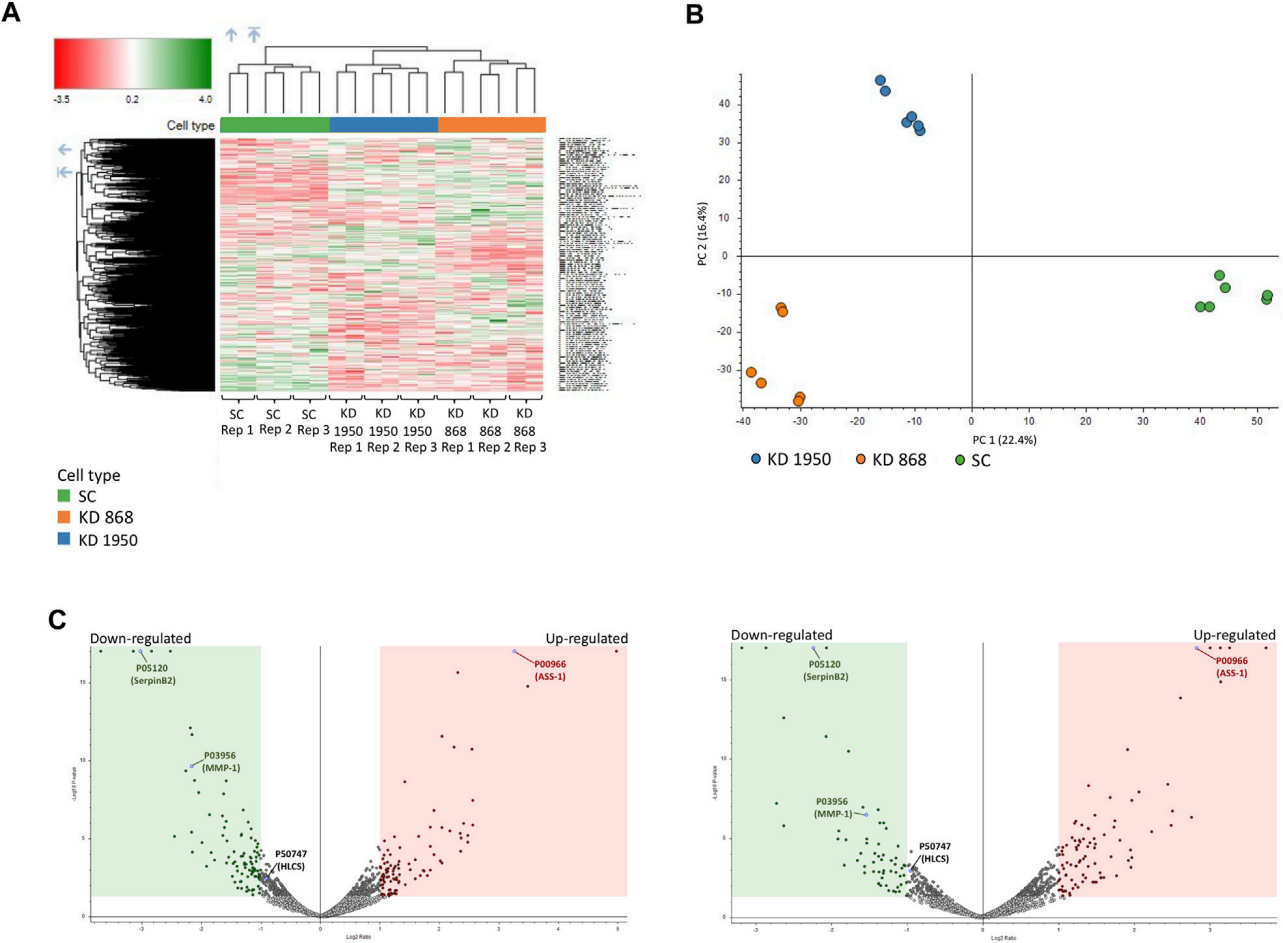
Proteomic analysis of HLCS knockdown MDA-MB-231 cells. Three biological replicates (REP1, REP2, REP3) of scrambled (SC) and two HLCS knockdown cell lines (KD 868 and KD 1950), each with two technical replicates, were performed. **(A)** Heat map and hierarchical clustering of 5,306 proteins identified in all samples. Color intensity indicates protein expression level. Green and red is upregulation and downregulation, respectively. **(B)** PCA analysis of proteomics data set. PC scores are based on individual proteomic data between scrambled control and KD 868 and KD 1950 clones. **(C)** Volcano plot of protein expression in HLCS knockdown KD 868 (left panel) and KD 1950 (right panel). Each colored dot represents a protein that displays a large magnitude of fold change relative to the scrambled control cell line (*X*-axis) and statistical significance (*Y*-axis). Green dots represent downregulated proteins, red dots represent upregulated proteins, and grey dots represent the unchanged proteins relative to those of the scrambled control cell line. Green and red squares indicate significant protein abundance differences (*p* < 0.05) less than log_2_FC = −1 or greater than log_2_FC = 1, respectively. ASS1, HLCS, SerpinB2, and MMP1 are shown in blue dots.

Two previously generated independent HLCS knockdown MDA-MB-231 cell lines (KD 868 and KD 1950) were used for proteomic analysis. These two HLCS knockdown cell lines possessed approximately 70%–80% reduction of HLCS protein corresponding to 86, 82, and 72 kDa isoforms (Bailey et al., 2010) (Figure 1A) compared to the scrambled control cell line. The decreased HLCS expression in these two knockdown clones was accompanied by 40%–60% reduction of clonogenic growth (Figure 1B). To gain further insight into how suppression of HLCS expression impacted global cellular changes, proteomic analysis was performed on the knockdown cells using LC-MS. The proteomic study was performed on these two HLCS knockdown cell lines to eliminate the clonal bias compared to the scrambled control cell line. All LC-MS data were obtained from three biological replicates, each with two technical replicates. According to the 1% false discovery rate (FDR) criteria with at least one unique peptide (Zhang et al., 2018; Yang et al., 2019), 5,306 proteins were identified across samples. Figure 2A shows the heat map of expression profiles and hierarchical clustering analysis of 5,306 proteins identified in all samples. Principle component analysis of all samples revealed a distinct cluster of scrambled control and HLCS knockdown clones KD 868 and KD 1950, respectively. The greatest component of variation of the data (PC1, 22.4%) showed a clear separation between the scrambled control and the knockdown groups, whereas the distinct cluster-based PC2 (16.4%) separation indicated a variable biological response between HLCS knockdown clones, KD 868 and KD 1950 (Figure 2B). Figure 2C shows the volcano plots of differentially expressed proteins identified between control and each HLCS knockdown cell lines [control vs. KD 868 (left panel) and control vs. KD 1950 (right panel)], separately. With the cut-off threshold of minimal 2-fold change with *p*-value <0.05, 196 proteins were considered differentially expressed. Of this number, 95 proteins were downregulated (spots in green box), while 101 proteins were upregulated (spots in red box). For the KD 1950 cell line, 151 proteins were differentially expressed, with 59 proteins being downregulated (spots in green box) and 92 proteins being upregulated (spots in red box).

### Functional annotation of biological pathways associated with DEPs

**FIGURE 3:**
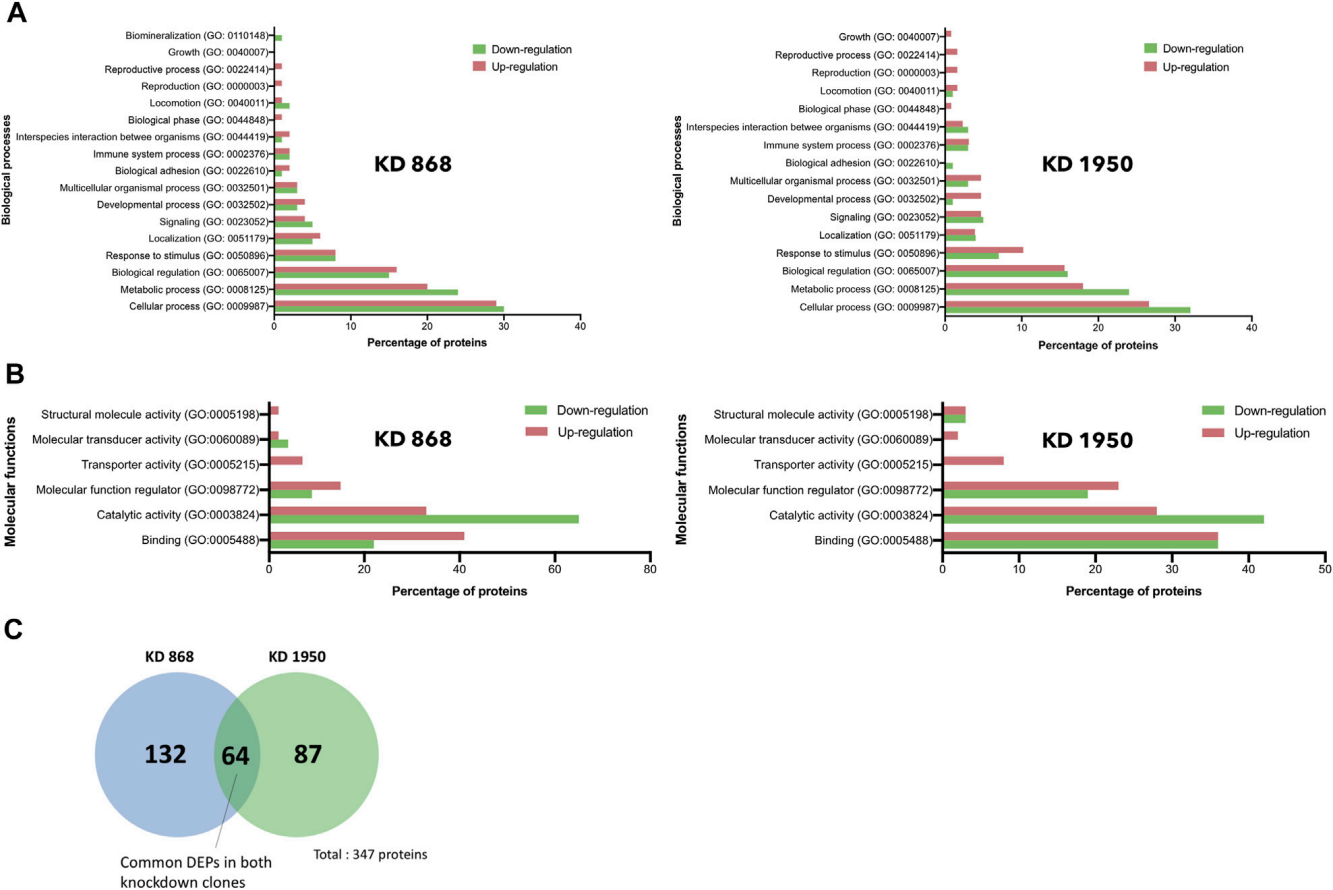
Gene ontology and pathway analysis of DEPs in HLCS knockdown MDA-MB-231 cells. Histograms depicting the percentage of pathway changes associated with HLCS knockdown in **(A)** Biological processes and **(B)** Molecular functions in KD 868 (left panel) and KD 1950 (right panel) clones. Green boxes indicate downregulated proteins, and red boxes indicate upregulated proteins. **(C)** Venn diagram showing distinct and common DEPs in both HLCS KD clones 868 and 1950.

To gain further insights into the biological response in HLCS knockdown, the functional annotation of the DEPs in each HLCS knockdown cell line was performed using the GO annotation tool. Based on the biological processes classification, the top three biological response changes associated with the downregulation of DEPs in the KD 868 clone were cellular process (GO:0009987, 30%), metabolic process (GO:0008125, 24%), and biological regulation (GO:00650007, 15%), with the other individual processes accounting for less than 10% (Figure 3A, left panel). Likewise, most changes associated with the downregulation of DEPs in the KD 1950 clone were cellular process (32%), metabolic process (24%), and biological regulation (16%), with the other processes listed similar to those of the KD 868 clone (Figure 3A, right panel). Regarding the upregulated proteins, the three most biological changes associated with the KD 868 clone were cellular process (GO: 0009987, 29%), metabolic process (GO: 0008152, 20%), and biological regulation (GO: 0065007, 16%). For the KD 1950 clone, the upregulated proteins were associated with cellular process (27%), metabolic process (18%), and biological regulation (16%).

According to the molecular function classification, the downregulated proteins in both HLCS knockdown clones were mostly involved in catalytic activity (GO:0003824; 65% in KD 868 (Figure 3B, left panel), and 42% in KD 1950 (Figure 3B, right panel), followed by binding activity (GO:0005488, 22% in KD 868, and 36% in KD 1950), and molecular function regulator (GO:0098772, 9% in KD 868, and 19% in KD 1950). However, the molecule transducer activity and structural molecule activity were molecular functions that exclusively related to the downregulated proteins in KD 868 and KD 1950, respectively. In contrast, the upregulated proteins in both knockdown clones were predominantly related to the binding activity (GO:0005488; 41% in KD 868, and 36% in KD 1950), followed by catalytic activity (GO:0003824; 33% in KD 868, and 28% in KD 1950), and molecular function regulator (GO:0098772; 15% in KD 868, and 23% in KD 1950).

### Common DEPs: MMP1, SerpinB2, and ASS1

To see whether the affected pathway changes in both HLCS knockdown cell lines were mediated through common proteins, the Venn diagram of DEPs of both knockdown clones was generated (Figure 3C). Both HLCS knockdown clones shared 64 common DEPs, with 23 being downregulated and 41 being upregulated. The list of these upregulated and downregulated and common proteins and their associated biological processes are shown in Table 1 and Table 2, respectively. According to the candidate proteins shown in Table 1, suppression of HLCS expression appears to be associated with increased programmed cell death, as shown by the increased expression of proteins involved in apoptosis [Apoptotic protease-activating factor 1 (APAF1)], immunogenic cell death [caspase-1 (CASP1)], autophagy [stimulator of interferon genes protein, (STING1)] and increased expression of proteins that possess tumor suppressor function such as tetraspanin-6 (TSPAN6), mitogen-activated protein kinase 6 (MAPK6), protein sprouty homolog 2 (SPRY2), HAUS augmin-like complex subunit 4 (HAUS4). The other common DEPs include those involved in metabolic processes, transcriptional regulation, cellular transport. Regarding the downregulated DEPs (Table 2), these proteins were associated with cell motility, such as myosin ID (MYO1D), leupaxin (LPXN), Interleukin-1β (ΙL-1β), plastin-2 (LCP1), transglutaminase 2 (TGM2), metallothionein 1X (MT1X), small p97/VCP-interacting protein (SVIP), and peroxiredoxin-like 2A (PRXL2A). The other affected biological processes include metabolic processes, cellular organization and biogenesis, and DNA-binding proteins.

**TABLE 1:** Abundance ratios (Log2) of upregulated proteins that are common in both knockdown clones relative to scrambled control cells.

Accession	Protein abbreviation	Protein name	KD 868			KD 1950	
			Fold change (Log2)		*p*-value (-Log10)	Fold change (Log2)	*p*-value (-Log10)
		***Regulation of biological process***					
C9JLV4	APAF1	Apoptotic protease-activating factor 1	2.0		3.6	1.6	2.5
D3DSM0	ITGB2	Integrin beta	1.3		1.7	2.0	3.8
H7C224	IRAK1	Interleukin-1 receptor-associated kinase 1	2.4		4.2	1.8	2.6
O43597	SPRY2	Protein sprouty homolog 2	1.2		1.7	1.8	4.9
O43657	TSPAN6	Tetraspanin-6	2.4		5.3	1.9	3.6
P29466	CASP1	Caspase-1	1.1		3.0	1.1	3.5
Q10589	BST2	Bone marrow stromal antigen 2	2.3		15.7	1.9	10.6
Q16659	MAPK6	Mitogen-activated protein kinase 6	1.3		2.7	1.2	3.3
Q86WV6	STING1	Stimulator of interferon genes protein	2.4		6.0	1.5	2.2
Q9H6D7	HAUS4	HAUS augmin-like complex subunit 4	2.2		10.9	1.4	4.5
Q9H9A7	RMI1	RecQ-mediated genome instability protein 1	1.2		1.1	1.5	1.5
Q9NQ25	SLAMF7	SLAM family member 7	2.0		3.4	2.5	5.8
		***Metabolic process***					
P00966	ASS1	Argininosuccinate synthase 1	3.3		17.0	2.8	17.0
P04114	APOB	Apolipoprotein B-100	1.0		1.5	1.3	2.7
P14618-2	PKM1	Isoform M1 of Pyruvate kinase PKM	1.1		2.7	1.7	7.6
P17900	GM2A	Ganglioside GM2 activator	1.9		5.7	2.1	7.9
Q8WV93	AFG1L	AFG1-like ATPase	1.3		2.2	1.0	1.7
Q9NXS2	QPCTL	Glutaminyl-peptide cyclotransferase-like protein	2.5		4.8	2.0	3.2
U3KPX1	ACYP2	Acylphosphatase	1.2		2.5	2.6	13.8
		***Transcriptional regulation***					
A0A0U1RRL5	ELF1	ETS-related transcription factor Elf-1	1.8		3.0	2.5	6.7
M0R3F3	MED29	Mediator of RNA polymerase II transcription subunit 29	1.7		3.0	1.1	1.6
O60907	TBL1X	F-box-like/WD repeat-containing protein	2.6		7.4	1.2	1.8
O95365	ZBTB7A	Zinc finger and BTB domain-containing protein 7A	1.2		1.5	1.6	2.5
Q16594	TAF9	Transcription initiation factor TFIID subunit 9	1.7		2.6	3.1	14.9
Q5JXX2	MORF4L2	Mortality factor 4-like protein 2	1.9		6.8	1.1	2.7
Q8IWI9	MGA	MAX gene-associated protein	1.8		2.6	2.8	6.3
Q969E4	TCEAL3	Transcription elongation factor A protein-like 3	1.1		4.3	1.1	4.5
		***Protein involved in transport***					
H3BQV3	COG8	Conserved oligomeric Golgi complex subunit 8	1.3		1.5	1.1	1.7
K7EPV6	SLC44A2	Choline transporter-like protein 2	2.5		10.7	1.7	6.1
P27105	STOM	Stomatin	1.0		2.6	1.3	4.9
Q13303	KCNAB2	Voltage-gated potassium channel subunit beta-2	1.7		5.1	1.2	3.3
Q9BZI7	UPF3B	Regulator of nonsense transcripts 3B	2.2		5.5	1.7	3.8
Q9Y6M7-7	SLC4A7	Isoform 7 of Sodium bicarbonate cotransporter 3	1.2		1.7	1.2	3.2
Q9Y6M9	NDUFB9	NADH:ubiquinone oxidoreductase subunit B9	1.1		3.0	1.3	4.8
		***Metal-ion binding***					
E7EV05	ZFAND2B	AN1-type zinc finger protein 2B	1.9		4.0	3.1	17.0
F8WCD0	RNF149	E3 ubiquitin-protein ligase RNF149	1.6		2.4	1.5	2.2
P33764	S100A3	Protein S100-A3	1.8		4.5	3.0	17.0
		***Transmembrane protein***					
A0A075B6H3	TMCO4	Transmembrane and coiled-coil domain-containing protein 4	1.2		1.5	1.5	2.8
		***Receptor***					
A0A1B0GVW0	ATP6AP2	Renin receptor	1.2		2.7	1.3	3.5
		***Unknown function***					
O60732	MAGEC1	Melanoma-associated antigen C1	1.3		3.2	1.3	5.0
U3KQP1	-	Uncharacterized protein	1.6		3.6	1.6	5.2

**TABLE 2:** Abundance ratio (Log2) of downregulated proteins that are common in both knockdown clones relative to scrambled control cells.

Accession	Protein abbreviation	Protein name	KD 868		KD 1950	
			Fold change (Log2)	*p*-value (-Log10)	Fold change (Log2)	*p*-value (-Log10)
		***Biological process***				
J3QRN6	MYO1D	Myosin ID	−1.5	2.6	−1.6	3.2
O60711	LPXN	Leupaxin	−2.8	17.0	−1.3	6.0
P01584	IL1B	Interleukin-1 beta	−1.1	1.7	−1.6	2.8
P03956	MMP1	Interstitial collagenase	−2.2	9.6	−1.5	6.5
P05120	SERPINB2	Plasminogen activator inhibitor 2	−3.0	17.0	−2.2	17.0
P13796	LCP1	Plastin-2	−2.5	17.0	−2.9	17.0
P21980	TGM2	Transglutaminase 2	−1.3	6.8	−1.8	10.5
P80297	MT1X	Metallothionein 1X	−1.3	3.4	−2.6	12.6
Q8N668	COMMD1	Copper metabolism domain containing 1	−1.6	3.4	−1.2	2.9
Q8NHG7	SVIP	Small VCP/p97-interacting protein	−1.6	4.9	−1.4	3.9
Q9BRX8	PRXL2A	Peroxiredoxin-like 2A	−1.3	4.0	−1.6	7.0
		***Metabolic process***				
A0A087WYS9	SURF1	SURF1-like protein	−1.1	3.1	−1.1	3.1
M0QXB5	ETHE1	Persulfide dioxygenase ETHE1, mitochondrial	−1.6	6.1	−1.4	6.8
P36269	GGT5	Glutathione hydrolase 5 proenzyme	−1.2	2.7	−1.5	4.7
Q16719	KYNU	Kynureninase	−3.1	17.0	−2.1	11.4
Q96AD5	ATGL	Adipocyte triglyceride lipase	−1.6	5.2	−1.4	4.6
Q96GK7	FAHD2A	Fumarylacetoacetate hydrolase domain-containing protein 2A	−2.2	12.1	−2.1	17.0
		***Cell organization and biogenesis***				
P0CG12	CHTF8	Decreased expression in renal and prostate cancer protein	−1.3	3.1	−1.0	2.2
Q96AY3	FKBP10	Peptidyl-prolyl cis-trans isomerase FKBP10	−2.0	7.9	−1.3	3.6
		***DNA-binding protein***				
Q96KM6	ZNF512B	Zinc finger protein 512B	−1.9	3.2	−1.3	2.0
Q9BQ70	TCF25	Transcription factor 25	−2.2	5.4	−1.9	5.5
		***Unknown function***				
P43363	MAGEA10	Melanoma-associated antigen 10	−3.7	17	−3.2	17.0
Q9NS25	SPANXB1	Sperm protein associated with the nucleus on the X chromosome B1	−2.1	8.7	−1.5	5.0

**FIGURE 4:**
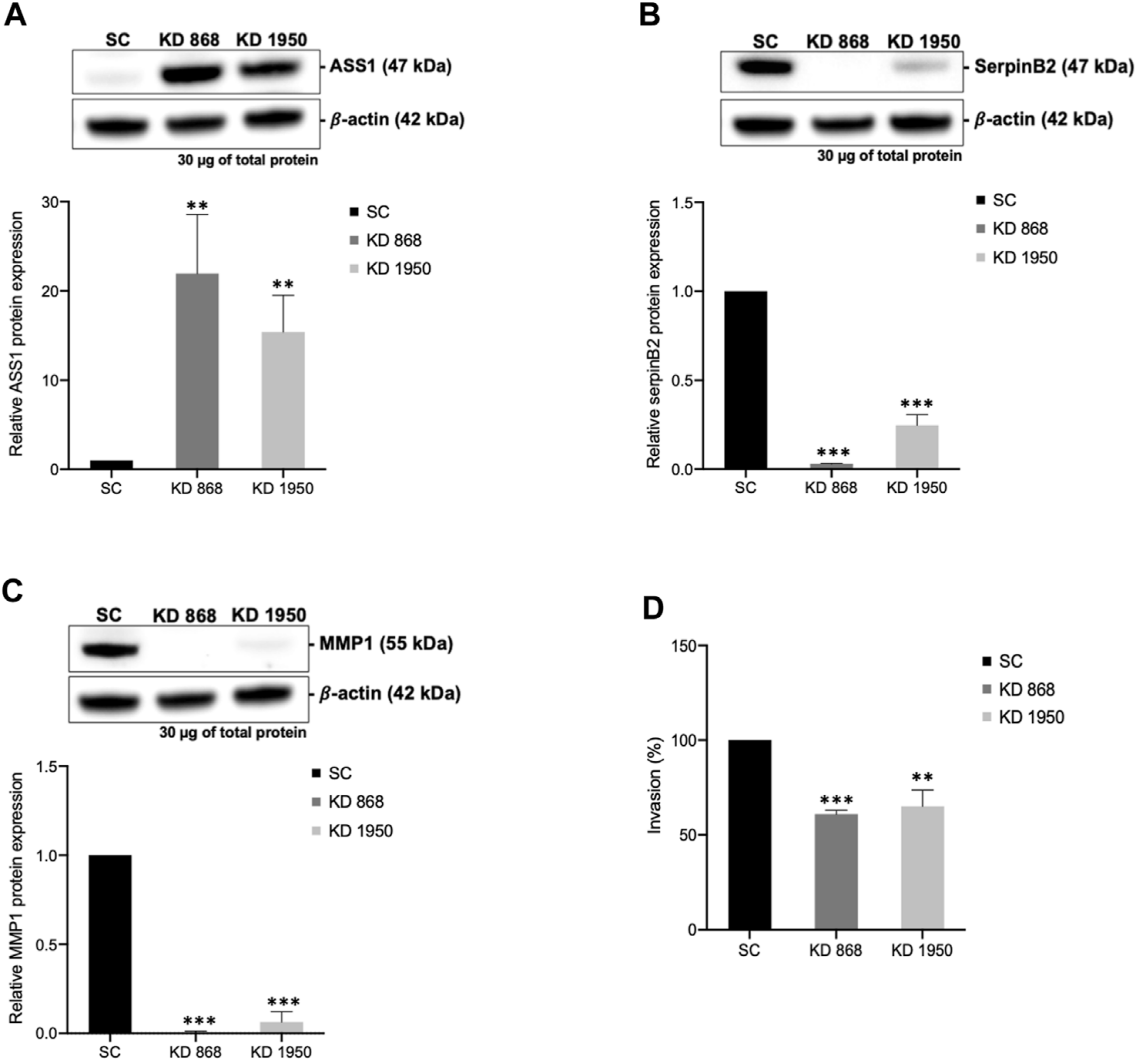
The marked induction of ASS1 and reduction of SerpinB2 and MMP1 expression in HLCS knockdown cells. Western blot analysis of ASS1 **(A)**, SerpinB2 **(B)**, and MMP1 **(C)**, and their expression levels relative to those of scrambled control cell line (bottom panel). **(D)** *In vitro* invasion assay of HLCS knockdown clones KD 868 and KD 1950. The number of invaded cells was relative to that of the scrambled control cell line, which was arbitrarily set as 100. **p < 0.01, ***p < 0.001.

To validate some of the candidate proteins shown in both Tables 1, 2, some candidates from both upregulated proteins and downregulated which showed the most significant fold changes, including argininosuccinate synthase 1 (ASS1) (log_2_ + 3.3 and + 2.8-fold in KD 868 and KD 1950 cells, respectively), plasminogen activator inhibitor 2 (SerpinB2) (−3.0 and −2.2-fold), interstitial collagenase/matrix metalloproteinase-1 (MMP1) (−2.2 and −1.5-fold) were validated by Western blot analysis. ASS1, one of the urea cycle enzymes, catalyzes the condensation of citrulline and L-aspartate to form argininosuccinate, and this enzyme is downregulated in many types of cancer (Hajaj et al., 2021). SerpinB2 has been shown to regulate stroma remodeling and metastasis (Harris et al., 2017), and MMP1 is one of the metalloproteases involved in cell invasion (Liu et al., 2012). As shown in Figure 4A, ASS1 expression level was extremely low in the scrambled control cell line, while it was markedly increased in both KD 868 and KD 1950 cell lines (*p* = 0.005 and *p* = 0.004, respectively). SerpinB2 expression was reduced by 95% in the KD 868 and was reduced by 75% in KD 1950 (*p* < 0.001) (Figure 4B). Similar to SerpinB2, MMP1 expression level was barely detectable in KD 868 and decreased by approximately 94% (*p* = 0.00001) in KD 1950 (Figure 4C). The marked reduction of MMP1 was accompanied by 40% decrease of invasion in both HLCS knockdown clones (Figure 4D).

## Discussion

We have previously shown that suppression of HLCS expression lowers biotinylation of carboxylases accompanied by impaired growth, migration and invasion, and cell cycle arrest in both luminal subtype (MCF-7) and triple-negative subtype (MDA-MB-231) breast cancer cell lines (Siritutsoontorn et al., 2022). Although these phenotypic defects were solely attributed to the lowered carboxylase activities, how reduced carboxylase activities perturb the biological and metabolic processes is largely unknown, leading to growth retardation.

### Holocarboxylase synthetase knockdown promotes program cell death and tumor suppressor proteins

GO analysis indicates that HLCS knockdown mainly affects cell death and other biological processes associated with cell motility and metabolic pathways. Regarding cell death, several proteins implicated in cell death pathways were increased—for example, the APAF1, CASP1, and STING1 (Table 1). APAF1 is a core component of the apoptosome, essential for caspase-9 activation (Pop et al., 2006). Increased APAF1 expression is well-described in many types of cancer exposed to chemotherapeutic drugs (Yuan et al., 2019; Xiang et al., 2020; Bakhshoudeh et al., 2021). CASP1 is a pro-inflammatory cytokine that cleaves pro-ΙL-1β, and pro-IL-18 to their active forms. This activity is essential for pyroptosis, a cell death process triggered by inflammation. A recent study shows that the chemotherapeutic drug, cisplatin, induces CASP1-mediated pyroptosis in MDA-MB-231 cells (Yan et al., 2021). CASP1-deficient mice showed enhanced colon epithelial and tumor proliferation, indicating its role as a tumor-suppressor protein (Hu et al., 2010). STING1, an ER-bound protein, is crucial for autophagy and programmed cell death (Zhang et al., 2021a). A recent study shows that Eribulin, an anti-cancer drug used to treat breast cancer and liposarcoma, triggers cancer cell death via the STING1-dependent signaling axis (Fermaintt et al., 2021; Takahashi-Ruiz et al., 2022), indicating its role as a cell-death-promoting factor. Upregulation of these pro-apoptotic proteins may be induced in response to HLCS suppression.

The reduced clonogenic growth of MDA-MB-231 herein may be attributed to the upregulation of some tumor suppressor proteins such as TSPAN6, MAPK6, SPRY2, HAUS4, stomatin (STOM) and conserved oligomeric golgi complex subunit 8 (COG8) (Table 1). TSPAN6, a tetraspanin family member, is a tumor suppressor protein that interferes with TGF-α signaling in colorectal cancer (Andrijes et al., 2021). Similarly, MAPK6 or ERK3, an atypical MAPK member, controls cell growth (Chen et al., 2000). A recent study shows that MAPK6/ERK3 exerts its tumor suppressor function by inhibiting cell cycle progression (Julien et al., 2003) and proliferation and invasion of melanoma (Chen et al., 2019a). SPRY2, a receptor tyrosine kinase inhibitor, has been shown to interfere with oncogenic signaling by antagonizing the ERK signaling pathway (Fong et al., 2006). Overexpression of SPRY2 in several types of cancer inhibits their growth and progression (Kawazoe and Taniguchi, 2019). HAUS4 is part of the HAUS augmin-like complex, which contributes to mitotic spindle assembly, maintenance of chromosome integrity, and completion of cytokinesis. Avelar et al., 2020 showed that elevated HAUS4 expression is associated with senescence and a tumor-suppressor gene. Stomatin, an integral membrane, regulates ion channels and transporter (Rahman et al., 2021). A recent study showed that stomatin suppresses proliferation and induces apoptosis by inhibiting Akt signaling in prostate cancer (Rahman et al., 2021; Sato et al., 2021). The decreased stomatin expression was associated with poor prognosis of prostate, breast, and non-small cell lung cancer patients, indicating its role as a tumor suppressor protein (Chen et al., 2016; An et al., 2019; Rahman et al., 2021). COG8 is the structural component of golgi-apparatus that functions in intracellular transport and glycosylation. COG8 level is negatively correlated with the survival of renal clear carcinoma patients (Zhang et al., 2021b).

### Holocarboxylase synthetase knockdown perturbs cellular function by down-regulating MMP1 and other cytoskeletal proteins

Extracellular matrix detachment is a fundamental process for cancer cell migration. This process involves metalloproteinase secretion and other proteases such as urokinase plasminogen activator. MMP1 is a member of matrix metalloproteinases, which degrades extracellular matrix during an invasion, while SerpinB2 regulates stromal remodeling, local invasion and metastasis (Valiente et al., 2014; Harris et al., 2017). Overexpression of MMP1 is associated with tumor invasion and metastasis (Chambers and Matrisian, 1997) and is highly expressed in triple-negative breast cancer (Wang et al., 2019). The decreased abundances of MMP1 and SerpinB2 in HLCS knockdown cells are consistent with impaired cell invasion and migration (Siritutsoontorn et al., 2022). The reduced cell invasion of HLCS deficient cells may also be associated with the decreased expression of cytoskeletal proteins, including SerpinB2, LPXN, MYO1D, LCP1, or their regulators, such as ΙL1B and TGM2 (Table 2). LPXN, a focal adhesion adaptor protein, is essential for cell adhesion and migration (Alpha et al., 2020). Recent studies show that LPXN is overexpressed in many cancers (Kaulfuss et al., 2008; Kaulfuss et al., 2015; Hou et al., 2018). Depletion of LPXN expression reduces prostate cell migration and invasion, while its overexpression promotes prostate cancer progression (Kaulfuss et al., 2009). MYO1D, a member of the myosin motor protein, controls cell movement (Diaz-Valencia et al., 2022) and promotes the growth and invasion of cancer cells (Amcheslavsky et al., 2018). MYO1D interacts with EGFR and contributes to oncogenesis (Ko et al., 2019; Mu et al., 2022). ΙL-1Β plays various roles in physiological processes, including inflammation, cell adhesion, migration, and angiogenesis (Rebe and Ghiringhelli, 2020). Although ΙL-1B is primarily produced from immune cells, it can be synthesized and secreted from several types of solid tumors (Jin et al., 1997; Wu et al., 2016), where it stimulates the expression of IL-6 and COX-2, which are promoting factor for breast cancer aggressiveness (Reed et al., 2009; Oh et al., 2016). Plastin-2 (LCP1), an actin-binding protein, regulates cytoskeleton movement. Plastin-2 is overexpressed in most types of cancer (Park et al., 1994; Tiedemann et al., 2019), supporting invasion and metastasis (Foran et al., 2006). TGM2 catalyzes the posttranslational modification of glutamine residue-bound proteins. TGM2 also plays a non-enzymatic function by interacting with fibronectin and integrin to stabilize ECM structure (Condello et al., 2022). TGM2 is overexpressed in many types of cancer (Agnihotri and Mehta, 2017), promoting proliferation, migration, and invasion (Wang et al., 2016).

In addition, HLCS suppression also downregulates some proteins involved in intracellular transport and stress response, i.e., copper metabolism domain containing 1 (COMMD1), SVIP, and PRXL2A. COMMD1 is a copper-binding protein with additional non-copper-related function (Weiskirchen and Penning, 2021). COMMD1 was elevated in lymphoma, and its level was correlated with poor prognosis (Taskinen et al., 2014). SVIP involves the ER-associated protein degradation of misfolded proteins. In cancer cells, SVIP was overexpressed in prostate cancer tissue, while its inhibition reduces migration and malignant transformation (Erzurumlu and Ballar, 2017). PRXL2A is a member of the peroxiredoxin antioxidant protein and functions in removing reactive oxygen species. PRXL2A is an essential protein in cancer stem cells that modulates redox status and maintains stemness properties. PRXL2A was overexpressed in oral squamous cell carcinoma and renal clear cell carcinoma, where high PRXL2A was associated with poor prognosis (Chen et al., 2019b; Ren et al., 2021). MT1X, a member of the metallothionein family, functions as a copper/zinc binding protein to maintain metal homeostasis and control oxidative stress and DNA damage (Krizkova et al., 2018). MT1X supports proliferation while inhibiting apoptosis and p53 expression in several types of cancer (Liu et al., 2018). MT1X was overexpressed in many cancers, including breast cancer (Si and Lang, 2018), and was a prognostic marker for invasive ductal carcinoma (Zhang et al., 2000) and renal carcinoma (Ding et al., 2022).

As HLCS knockdown cells showed marked reduction of invasion accompanied by reduced MMP1 expression, we checked whether these defective phenotypes were attributed to impaired epithelial-mesenchymal transition program and stemness of cancer cells. Western blot analysis of vimentin and Oct4 showed that the levels of these two proteins were unchanged, suggesting the impaired clonogenic growth and invasion were not due to the loss of EMT and stemness (Supplementary Figure S1).

### Holocarboxylase synthetase suppression perturbs the expression of metabolic enzymes

In addition to the changes in cellular processes, HLCS knockdown also affects metabolic pathways related to both glycolysis and mitochondrial metabolism. One of the most apparent changes is the increased expression of ASS1, suggesting that the urea cycle may be compromised in response to HLCS knockdown. The urea cycle is not only a cycle that disposes ammonium from amino acid catabolism, but it is also linked to the TCA cycle through aspartate. Transamination of oxaloacetate produces L-aspartate, which enters the urea cycle, where it condenses with L-citrulline to form argininosuccinate mediated by ASS1. Because L-aspartate is an essential nitrogen donor for purine biosynthesis, modulation of ASS1 expression would determine the cellular aspartate pool, which supports nucleotide synthesis during proliferation. ASS1 also plays a non-metabolic role by suppressing Akt signaling and attenuating cancer growth (Miyamoto et al., 2017). Growing evidence indicates that many cancers inactivate urea cycle enzymes, including ASS1 (Rabinovich et al., 2015; Lee et al., 2018b), so the L-aspartate is available for nucleotide synthesis. Ectopic expression of the ASS1 gene in cancer cells inhibits their growth, indicating its role as a tumor suppressor enzyme (Khare et al., 2021; Kim et al., 2021). The marked increase of ASS1 expression in HLCS knockdown cells suggests that urea cycle activity may be increased as previously reported in hepatocytes of liver-specific PC knockout mice (Cappel et al., 2019). The increased ASS1 expression can also affect immediate downstream reactions in the urea cycle, i.e., the conversion of argininosuccinate to arginine catalyzed by arginosuccinate lyase. Because arginine is a precursor of nitric oxide, a chief mediator of inflammatory cytotoxicity, elevated arginine levels due to increased ASS1 would enhance NO production (Kim et al., 2021). Interestingly, PC KO pancreatic cells showed elevated urea cycle activity accompanied by increased arginine and NO production (Fu et al., 2020). The increased NO, in turn, promotes oxidative stress in PC KO pancreatic beta cells, contributing to cell death (Fu et al., 2020). Because HLCS regulates PC activity through biotinylation, it is not unexpected to see a similar perturbation of urea cycle activity in both HLCS and PC-deficient cells. However, it is still unclear how suppression of HLCS expression induces ASS1 expression. Although the expression of ASS1 was markedly increased in MDA-MB-231 cells, we did not see the same response in HLCS knockdown MCF-7 cells (Supplementary Figure S2). However, this outcome was anticipated as the basal level of ASS1 expression in MCF-7 cell line was already high. The lack of ASS1 change in HLCS knockdown MCF-7 cells may recapitulate the early stage of cancer, where ASS1 may not be essential during early stage of tumor progression. This is consistent with previous studies demonstrating that not all breast cancer subtypes inactivate ASS1 expression to enhance their oncogenic property (Qiu et al., 2014; Zou et al., 2021). Qiu et al. (2014) showed that highly invasive MDA-MB-231 cells regulate their oncogenic property by down-regulating ASS1 expression, but this is not the case for low invasive cell lines, i.e., MCF-7 and T47D, which possess much higher levels of endogenous ASS1. This indicates that the low and high invasive breast cancer cell lines regulate their oncogenic potentials via ASS1 differently (Qui et al., 2014). Qui et al. also showed that breast cancer patients with stage 0-I possess higher ASS1 expression, while those with advanced stage (III and IV) possess considerably lower ASS1 levels, suggesting the inactivation of ASS1 expression may occur during the transition from low to advanced stages of breast cancer.

In addition to the urea cycle, HLCS knockdown cells showed increased pyruvate kinase M1 (PKM1) isoform expression. While PKM1 is a highly active isoform, most cancers expressed PKM2 rather than PKM1 to promote Warburg’s effect. The increased PKM1 level in HLCS knockdown could attenuate cancer growth, as reported in prostate adenocarcinoma, where inhibiting receptor tyrosine kinase signaling induces the expression of PKM1 and attenuates the growth of glioblastoma (Yang et al., 2022). The tumor suppressor role of PKM1 was also reported in breast cancer (Choksi et al., 2021) and prostate adenocarcinoma (Davidson et al., 2022).

HLCS knockdown also negatively affects lipid metabolism by altering expression of adipocyte triglyceride lipase (ATGL) (Table 2) and apolipoprotein B-100 (APOB) (Table 1). ATGL catalyzes the hydrolysis of triglycerides to free fatty acid and diacylglycerol. ATGL was overexpressed in several types of cancer, suggesting that fatty acid catabolism is essential to support the growth of tumors (Iftikhar et al., 2021; Yin et al., 2021; Zhang et al., 2022). APOB, a component of low-density lipoprotein, functions in triglyceride transport. APOB has recently been reported to play a role as tumor suppressor protein. The loss of APOB expression in several types of cancers is also associated with the upregulation of oncogenes and poor prognosis (Lee et al., 2018a; He et al., 2022). Mechanistically, overexpression of APOB in MDA-MB-231 cells inhibits their growth and invasion by depleting the lipid supply to the breast cancer cells (Ben Hassen et al., 2020).

The reduced growth of HLCS knockdown cells may result from the perturbation of mitochondrial oxidative phosphorylation, causing mitochondrial stress and apoptosis via downregulation of SURF1-like protein (SURF1), persulfide dioxygenase (ETHE1), and NADH: ubiquinone oxidoreductase subunit B9 (NDUFB9) (Tables 1, 2). SURF1 encodes an assembly factor of cytochrome oxidase of respiratory complex IV. The reduced expression of SURF1 can potentially impair cytochrome C oxidase activity, accelerating mitochondrial apoptosis in response to oxidative stress (Pequignot et al., 2001; Schull et al., 2015). ETHE1, a mitochondrial enzyme, detoxifies hydrogen persulfide, a harmful agent for cytochrome C oxidase. A recent study showed that ETHE1 is overexpressed in colorectal cancer, promoting aerobic glycolysis and mitochondria biogenesis (Witherspoon et al., 2019). Depleting ETHE1 expression induces oxidative stress, which aligns with the increased apoptosis of HLCS knockdown cells. NDUFB9 is a component of NADH dehydrogenase in the respiratory chain complex I. NDUFB9 was downregulated in highly metastatic breast cancer and silencing its expression in MDA-MB-231 cells promotes proliferation, migration, and invasion (Li et al., 2015). This inhibitory effect of NDUFB9 on tumor growth may be attributed to enhanced aerobic glycolysis in cancer cells (Li et al., 2015). The upregulation of NDUFB9 expression may be partly responsible for restricting MDA-MB-231 cell growth in response to HLCS suppression. The ganglioside GM2 activator (GM2A) activator protein is a specific glycolipid transport that binds to ganglioside GM2 and facilitates degradation. GM2A is associated with a programmed-cell death via interaction with tumor-necrosis receptor 1 receptor (Mahata et al., 2015). The increased abundance of GM2 activator may be attributed to the increased apoptosis in HLCS knockdown cells.

Kynureninase (KYNU) is an enzyme that catalyzes the reversible conversion of kynurenine to anthranilate and L-alanine. Kynurenine serves as the immunomodulant that protects cells against strong immune reactions. A recent study indicates that tumor cells hijack the kynurenine pathway to promote their growth and protect them from immune surveillance (Venkateswaran and Conacci-Sorrell, 2020). Kynurenine supports tumor growth by acting as the ligand for the aryl hydrocarbon receptor-transcription factor that activates oncogenic growth. Regarding immunomodulation, kynurenine suppresses T-lymphocyte proliferation by modulating their metabolism (Siska et al., 2021). Kynurenin was overexpressed in ductal and renal carcinoma, which confers ferroptosis-cell death (Liu et al., 2023).

**FIGURE 5:**
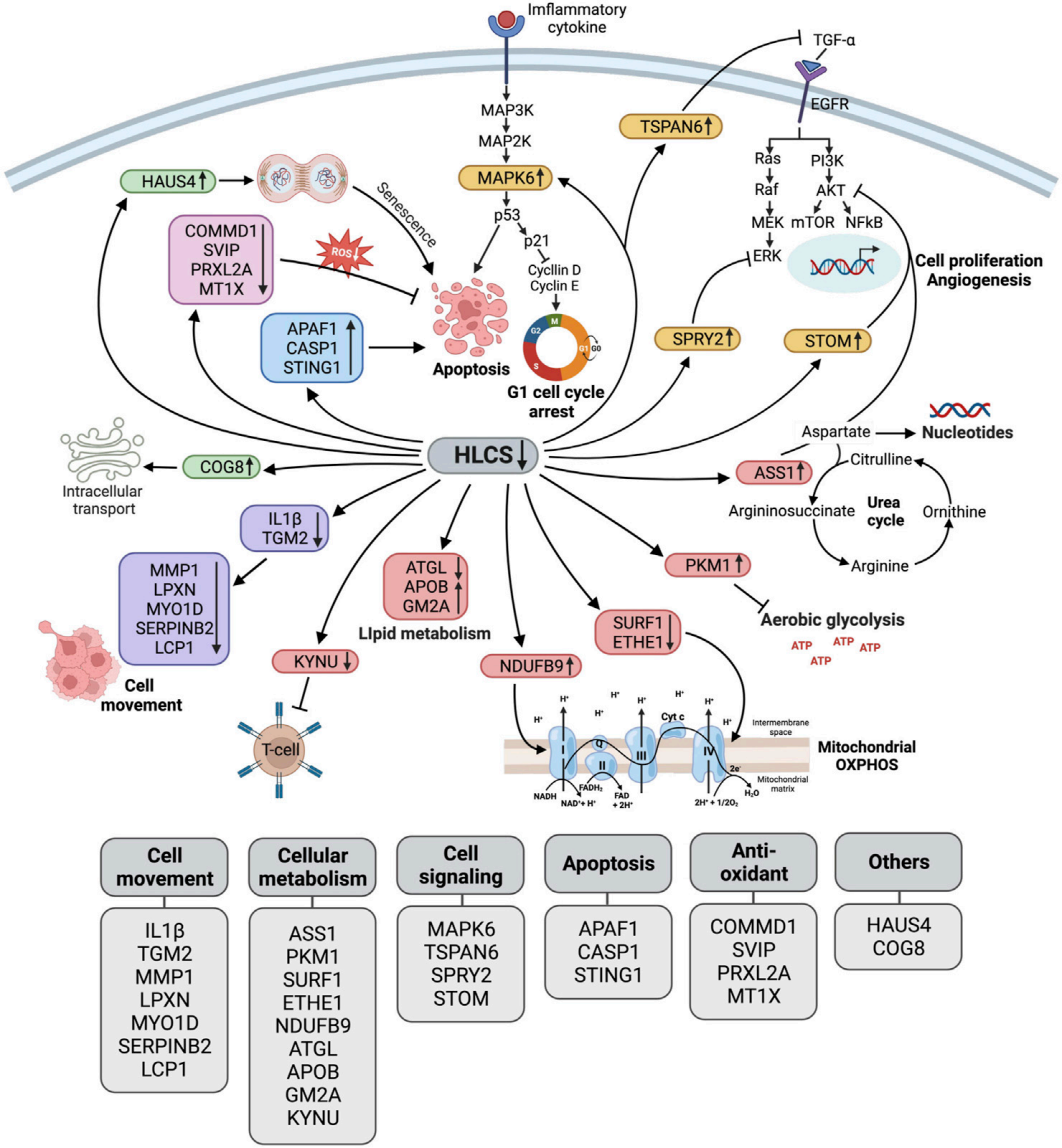
Schematic diagram illustrating the biological pathway changes in response to HLCS knockdown. Based on proteomic data, HLCS suppression promotes proteins involved in cell death (APAF1, CASP1, STING1 as shown in the blue box) and senescence (HAUS4 and COG8 as shown in the green box) while decreasing expression of antioxidant proteins (COMMD1, SVIP, PRXL2A, and MT1X as shown in the pink box), oncogenic signaling (MAPK6, TSPAN6, SPRY2, and STOM as shown in the yellow box).These changes probably contribute to growth restriction, oxidative stress, and cell death. Downregulation of the cytoskeleton and its regulatory proteins (IL1Β, TGM2, MMP1, LPXN, MYO1D, SerpinB2, and LCP1, as shown in purple) likely result in impaired migration and invasion. Depletion of HLCS perturbs metabolic pathways (shown in red box) via increased expression of ASS1, which regulates the aspartate pool available for nucleotide synthesis, and PKM1, which antagonizes aerobic glycolysis. Other metabolic disruptions include the downregulation of proteins that support respiratory chain activities (SURF1, ETHE1, and NDUFB9), lipid metabolism (ATGL, APOB, and GM2A), and attenuating kynurenine metabolism (KYNU).

Figure 5 summarizes the biological changes in response to HLCS knockdown. As previously reported, these combined effects likely contribute to growth retardation, migration, invasion, cell cycle arrest, and apoptotic induction.

## Data Availability

The datasets presented in this study can be found in online repositories. The names of the repository/repositories and accession number(s) can be found in the article/Supplementary Material.
