# Supplementary material for: Proteomic analysis of holocarboxylase synthetase deficient-MDA-MB-231 breast cancer cells revealed the biochemical changes associated with cell death, impaired growth signaling, and metabolism
Source: Front Mol Biosci. 2024 Jan 11;10:1250423. doi: 10.3389/fmolb.2023.1250423 (PMC10812114; doi:10.3389/fmolb.2023.1250423)
Supplement: Supplementary file 1 [file DataSheet2.DOCX]

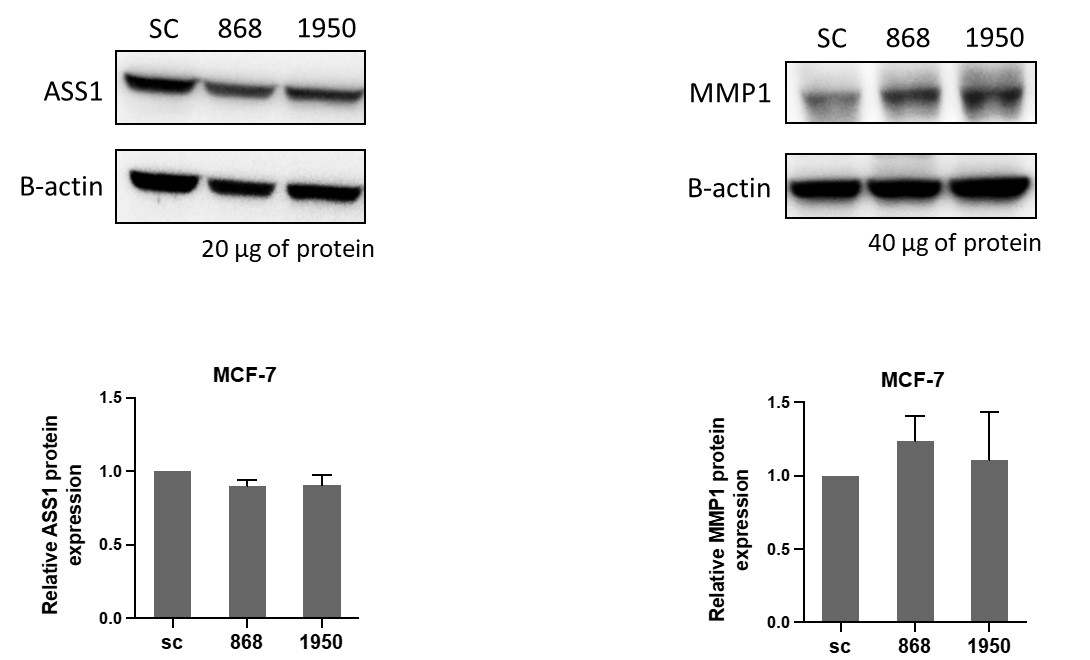


**Supplementary Figure 2**. Western blot analysis of ASS1 and MMP1 expression in HLCS knockdown MCF-7 cells (KD868 and KD1950). Representative Western blot analysis of ASS1 (left panel), and MMP1 (right panel), and their expression levels relative to those of scrambled control cell line (SC) (bottom panel). The data were obtained from three independent experiments.
