# Supplementary material for: Proteomic analysis of holocarboxylase synthetase deficient-MDA-MB-231 breast cancer cells revealed the biochemical changes associated with cell death, impaired growth signaling, and metabolism
Source: Front Mol Biosci. 2024 Jan 11;10:1250423. doi: 10.3389/fmolb.2023.1250423 (PMC10812114; doi:10.3389/fmolb.2023.1250423)
Supplement: Supplementary file 2 [file DataSheet1.DOCX]

**Supplementary Figure 1**. Western blot analysis of vimentin and Oct4 expression in scrambled control (SC) and two HLCS knockdown MDA-MB-231 cells (KD868 and KD1950). Representative Western blot analysis of Vimentin (left panel), and Oct4 (right panel), and their expression levels relative to those of scrambled control cell line (bottom panel). The results were obtained from three independent experiments.
